# Conjunctival Sarcoidosis Presenting as a Salmon‐Pink Lesion: A Lymphoma Mimicker

**DOI:** 10.1002/ccr3.70730

**Published:** 2025-07-31

**Authors:** Zahra Karjou, Kimia Alborzi, Sajjad Daneshyar

**Affiliations:** ^1^ Ophthalmic Research Center, Research Institute for Ophthalmology and Vision Science Shahid Beheshti University of Medical Sciences Tehran Iran; ^2^ Department of Neurology, School of Medicine Hamadan University of Medical Sciences Hamadan Iran

**Keywords:** conjunctival lesion, granulomatous inflammation, pathology, sarcoidosis

## Abstract

This case highlights an unusual presentation of conjunctival sarcoidosis that clinically mimicked lymphoma. It emphasizes the importance of histopathological evaluation and systemic workup in patients with atypical conjunctival lesions to ensure accurate diagnosis and appropriate management.

## 1 Introduction

Conjunctival lesions can exhibit a diverse range of clinical features, ranging from harmless growths to malignant tumors, often creating diagnostic difficulties. One specific manifestation is the “salmon‐pink” lesion, which is traditionally linked to conjunctival lymphoma [1]. However, similar appearances can also arise in inflammatory or granulomatous conditions, necessitating thorough evaluation to prevent misdiagnosis. Sarcoidosis is a chronic multisystem granulomatous disorder believed to stem from an exaggerated cellular immune response to various self‐antigens or non‐self‐antigens [2]. This condition is a multisystem granulomatous disease of unknown cause, most frequently impacting the lungs, lymph nodes, skin, and eyes. Ocular involvement can occur in as many as 25%–50% of cases, with anterior uveitis being the most common manifestation [3, 4]. Although less prevalent, conjunctival involvement may be the initial or sole indication of systemic disease. The classical characteristics of ocular sarcoidosis include large keratic precipitates (commonly referred to as mutton fat Kps), mild anterior chamber cells, tented peripheral anterior synechiae, posterior synechiae, nodules on the iris, and retrolental cells [5]. The most prevalent posterior segment manifestations are vitritis associated with snowballs or snow banking, retinal vessel vasculitis that mimics candle wax dripping, diffuse sarcoid granulomas, retinitis, and optic disc swelling. This case report emphasizes an atypical presentation of conjunctival sarcoidosis in a 50‐year‐old woman, who was initially thought to have conjunctival lymphoma, highlighting the necessity for histopathological analysis and comprehensive systemic evaluation in patients with unusual conjunctival lesions.

## 2 Case Presentation

**FIGURE 1:**
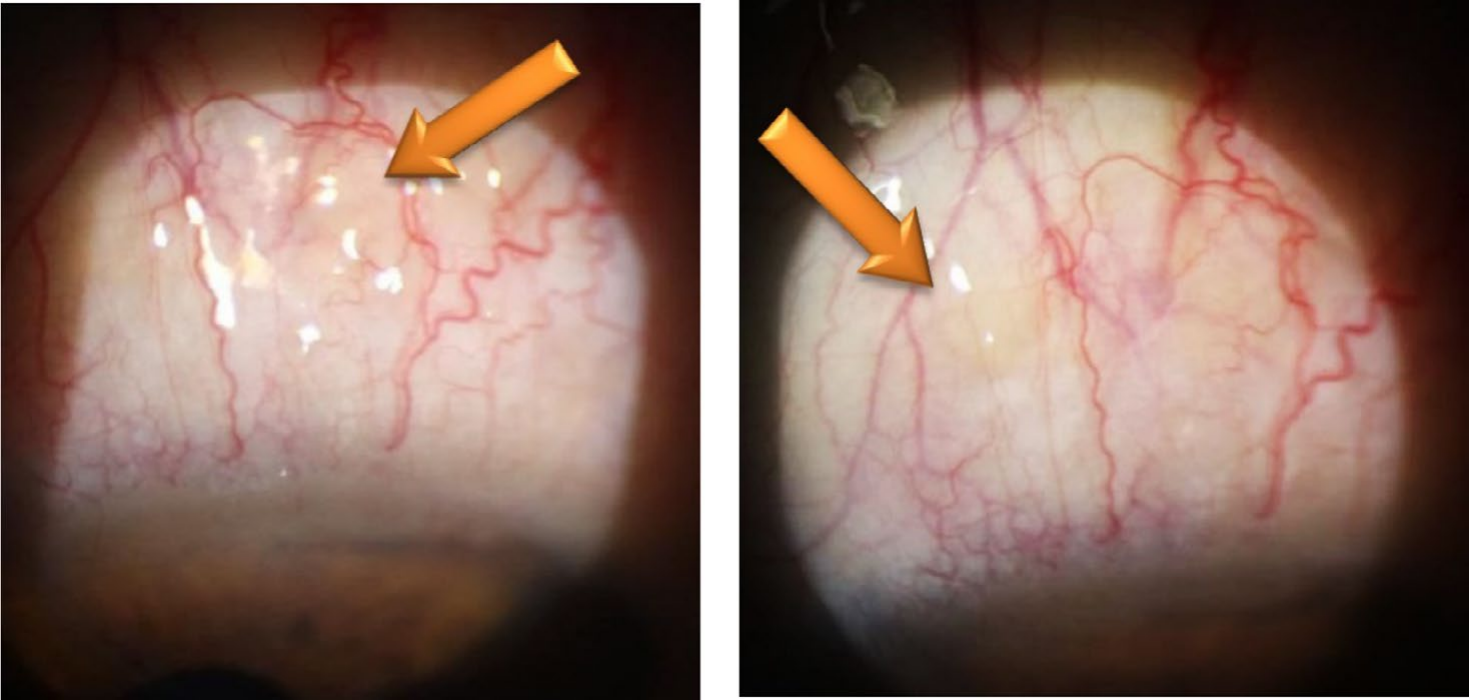
Diffuse prominent lesion of sharp Salmon Pink in the superior bulbar conjunctiva.

A 50‐year‐old female presented with a 4‐month history of foreign body sensation and ptosis in the left eye. She had undergone blepharoplasty surgery 3 years prior. On examination, the right eye showed no pathological findings. In the left eye, a diffuse, prominent, sharp‐edged salmon‐pink lesion measuring 3 mm was observed in the superior bulbar conjunctiva, just above the upper limbus (Figure 1). Visual acuity was 20/20, intraocular pressure measured 14 mmHg, and fundus examination was unremarkable.

## 3 Methods

**FIGURE 2:**
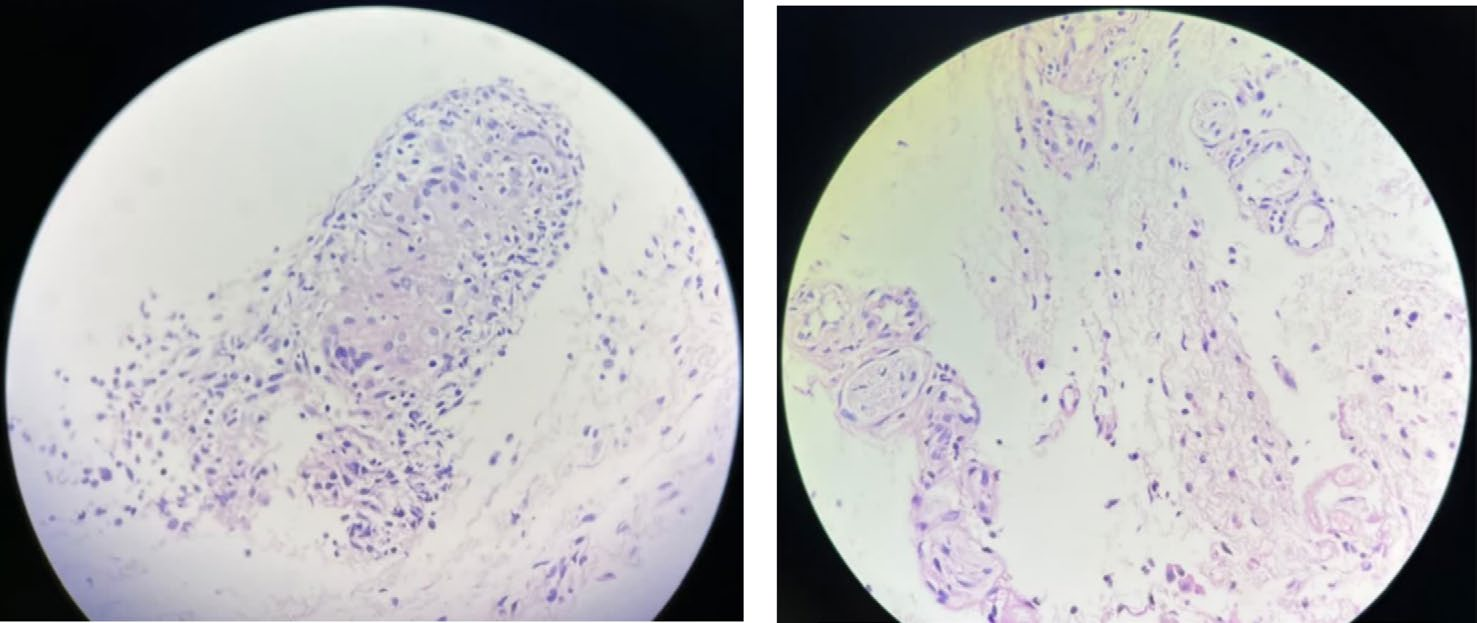
Granulomatous inflammation in the left conjunctival lesion consists of two pieces of creamy–grayish soft tissue measuring 0.5 × 0.3 × 2 cm.

Based on the clinical appearance of the lesion, conjunctival lymphoma was initially suspected. A biopsy was taken from the conjunctival lesion in the left eye, and histopathological analysis revealed granulomatous inflammation (Figure 2). Given the clinical findings, histopathological report showing granulomatous inflammation, and elevated serum angiotensin‐converting enzyme (ACE) levels (77 U/L; normal range: 8–52 U/L), a diagnosis of sarcoidosis was confirmed following rheumatology consultation. Chest CT scan showed no signs of pulmonary involvement, and further systemic evaluation revealed no evidence of extrapulmonary organ involvement. The patient was started on oral prednisolone and azathioprine, and complete resolution of the conjunctival lesions was observed within 1 month of treatment.

## 4 Conclusions

This case highlights an atypical presentation of sarcoidosis as a solitary conjunctival lesion, initially mimicking lymphoma. Diagnosis was confirmed through histopathology, elevated ACE levels, and rheumatological evaluation. It underscores the importance of considering sarcoidosis in conjunctival lesions and the need for a multidisciplinary approach to ensure accurate diagnosis and timely management, preventing systemic complications and improving outcomes.

## 5 Discussion

Sarcoidosis is a multisystem granulomatous disorder characterized by non‐caseating granulomas, which can affect virtually any organ system. Ocular involvement is a well‐documented manifestation of sarcoidosis, occurring in 25%–50% of patients with systemic disease [1, 2]. The presentation of ocular sarcoidosis can vary widely, ranging from anterior uveitis to conjunctival nodules, as seen in this case [3]. Conjunctival involvement, while less common, may be the initial or sole manifestation of the disease, as in the present case. The clinical appearance of a salmon‐pink conjunctival lesion often raises suspicion for conjunctival lymphoma, especially when it presents as a well‐circumscribed, painless mass. However, similar presentations may also occur in other conditions, including benign reactive lymphoid hyperplasia, amyloidosis, and granulomatous diseases such as sarcoidosis and tuberculosis. In our case, the initial suspicion was conjunctival lymphoma, but histopathology revealed non‐caseating granulomatous inflammation, leading to further systemic workup. Notably, systemic evaluation did not reveal any additional organ involvement, highlighting the possibility of isolated ocular sarcoidosis, a less common but documented presentation [4]. Conjunctival involvement in sarcoidosis, though less common than uveitis, is a recognized manifestation. Conjunctival nodules are typically asymptomatic but can present with foreign body sensation, redness, or ptosis, as observed in this case [5]. The differential diagnosis for such lesions includes infectious granulomas (e.g., tuberculosis), inflammatory conditions (e.g., granulomatosis with polyangiitis), and neoplasms (e.g., lymphoma or squamous cell carcinoma). Histopathological examination remains the gold standard for distinguishing these entities [6]. The diagnosis of sarcoidosis can be challenging, particularly in cases with isolated ocular involvement. The International Workshop on Ocular Sarcoidosis (IWOS) has established diagnostic criteria to aid clinicians in identifying ocular sarcoidosis [7, 8]. These criteria include both clinical findings (e.g., mutton‐fat keratic precipitates, trabecular nodules) and systemic investigations (e.g., elevated ACE levels, chest imaging). In this case, the combination of histopathological findings, elevated ACE levels, and rheumatological evaluation fulfilled the diagnostic criteria for sarcoidosis [9, 10]. This case underscores the importance of including sarcoidosis in the differential diagnosis of conjunctival lesions, particularly when the lesion mimics lymphoma. Histopathological confirmation remains essential for accurate diagnosis and guiding appropriate management. Furthermore, collaboration with rheumatology or internal medicine specialists is crucial to evaluate for systemic involvement and determine the need for systemic treatment or monitoring.

## Author Contributions

**Zahra Karjou:** conceptualization, project administration, validation. **Kimia Alborzi:** data curation, formal analysis, methodology, writing – original draft. **Sajjad Daneshyar:** data curation, writing – review and editing.

## Consent

Written informed consent was obtained from the patient to publish this report in accordance with the journal's patient consent policy.

## Conflicts of Interest

The authors declare no conflicts of interest.

## Data Availability

The data that support the findings of this study are available from the corresponding author upon reasonable request.
